# Human AP Endonuclease 1: A Potential Marker for the Prediction of Environmental Carcinogenesis Risk

**DOI:** 10.1155/2014/730301

**Published:** 2014-08-26

**Authors:** Jae Sung Park, Hye Lim Kim, Yeo Jin Kim, Jong-Il Weon, Mi-Kyung Sung, Hai Won Chung, Young Rok Seo

**Affiliations:** ^1^Environmental Health Research Department, National Institute of Environmental Research, 42 Hwangyeong-ro, Seo-gu, Incheon 404-708, Republic of Korea; ^2^School of Public Health, Seoul National University, 1 Gwanak-ro, Gwanak-gu, Seoul 151-742, Republic of Korea; ^3^Department of Life Science, Institute of Environmental Medicine, Dongguk University, 30 Pildong-ro 1-gil, Jung-gu, Seoul 100-715, Republic of Korea; ^4^Department of Safety Engineering, Dongguk University, Gyeongju, Gyeongbuk 780-714, Republic of Korea; ^5^Department of Food and Nutrition, Sookmyung Women's University, Cheong-ro 47-gil 100, Youngsan-gu, Seoul 140-742, Republic of Korea

## Abstract

Human apurinic/apyrimidinic endonuclease 1 (APE1) functions mainly in DNA repair as an enzyme removing AP sites and in redox signaling as a coactivator of various transcription factors. Based on these multifunctions of APE1 within cells, numerous studies have reported that the alteration of APE1 could be a crucial factor in development of human diseases such as cancer and neurodegeneration. In fact, the study on the combination of an individual's genetic make-up with environmental factors (gene-environment interaction) is of great importance to understand the development of diseases, especially lethal diseases including cancer. Recent reports have suggested that the human carcinogenic risk following exposure to environmental toxicants is affected by APE1 alterations in terms of gene-environment interactions. In this review, we initially outline the critical APE1 functions in the various intracellular mechanisms including DNA repair and redox regulation and its roles in human diseases. Several findings demonstrate that the change in expression and activity as well as genetic variability of APE1 caused by environmental chemical (e.g., heavy metals and cigarette smoke) and physical carcinogens (ultraviolet and ionizing radiation) is likely associated with various cancers. These enable us to ultimately suggest APE1 as a vital marker for the prediction of environmental carcinogenesis risk.

## 1. Introduction

Apurinic/apyrimidinic endonuclease/redox effector factor 1 (APE1/Ref-1, APEX1, here referred to as APE1) has multifunctions as a base excision repair enzyme and as a redox coactivator of a number of important transcription factors. Human APE1 protein working as a monomer is comprised of 318 amino acids and is divided into two different domains [1]. The N-terminal domain, including the nuclear localization signal (NLS) region, exerts the redox regulation activity, while the C-terminal domain is employed in the endonuclease activity at abasic site [2]. It is well known that these two domains operate independently, as explained in various reports about the mutation of specific amino acids such as Cysteine 65 (at the N-terminal end) and Histidine 309 (at the C-terminal end) in each domain [3, 4]. Unlike human APE1 protein, there is only one active function in* Escherichia coli* Xth and* Saccharomyces cerevisiae* APN2 which is APE1 endonuclease activity [5]. Human APE1 homology with other organisms includes the C-terminus which is highly conserved among various classes while the N-terminal domain is mostly conserved in mammal [6, 7].

APE1 is involved not only as key element of base excision repair and as a redox factor for regulation of transcription factors, but also as an RNA modulator and transcriptional repressor. On account of the multifunction of APE1 in humans, this protein is suggested as a crucial target in the pathology of cancers, neuronal diseases, aging, among others. Along with the APE1 related studies in pathology, previous research in our group demonstrated the correlation between environmental toxicants such as heavy metals and redox factors, which are important in the suppression of carcinogenesis [8–11]. Gene-environment interaction is regarded as an essential point to investigate diseases caused by environmental toxicants.

In this review, we begin with an exploration of various intracellular functions of APE1 which include DNA repair and redox regulation and continue to summarize the human pathologies related to APE1 alterations. Notably, we emphasize APE1 as a potential marker for risk prediction of environmental diseases induced by exposure to heavy metals, cigarette smoke toxicants, and radiation in terms of gene-environment interaction.

## 2. Multifunctions of APE1

**Figure 1:**
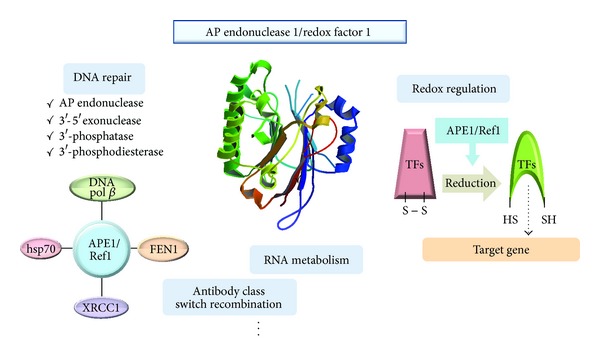
Multifunction of APE1/Ref1. APE1 has varied and independent functions and has an essential role in maintaining cellular stability. APE1 enzymatically restores damaged DNA bases and interacts with other proteins involved in DNA repair. APE1 regulates redox status of various transcription factors including AP-1 and p53 with its Cys residue. The crystal structure of APE1 (PDB ID : 1HD7) was identified by Beernink et al. [12].

A transacting protein, APE1, is considered an essential protein for maintaining cellular stability through various intracellular functions including the regulation of DNA repair and redox status (Figure 1). Since numerous authors have recently reviewed the extensive studies related to APE1's multifunction [13–16], our review focuses on the APE1's distinct functions.

### 2.1. APE1 in DNA Repair Mechanisms

#### 2.1.1. DNA Repair Activity of APE1

Base excision repair (BER), one of the DNA repair mechanisms, is crucial to maintenance of genomic stability by restoring damaged DNA bases. Through the BER pathway, an apurinic/apyrimidinic (AP) site is temporarily generated by glycosylase [17, 18] and can increase cytotoxicity and mutagenicity caused by blocking DNA replication [19]. APE1 recognizes the AP site and plays a vital role in initiation of BER as cleavage 5′-phosphodiester bonds at the AP site, generating a 3′-hydroxyl group and a 5′-2′-deoxyribose phosphate (dRP) group [20]. AP endonuclease activity of APE1 was demonstrated by Marenstein et al. [17] to show that the protein acts on the AP site in double-stranded DNA as well as single-stranded DNA suggesting it may act on each substrate during different nuclear states. Also, they showed that the efficiency of its activity was comparable in the two different forms of DNA.

A ubiquitously expressed protein, APE1, has been identified as carrying not only an AP endonuclease activity, but also other catalytic activities such as showing 3′-5′ exonuclease, 3′-phosphatase, and 3′-phosphodiesterase activity [21–24]. These additional activities excise 3′-end blocking groups which might induce genomic instability like mismatched bases. Although mammalian APE1 has weak activity of 3′-damage excision relative to other phyla (*E*.* coli* or yeast) [25–27], this activity is required to remove 3′-end blocking groups produced by radiation, ROS, or mammalian glycosylases NEIL1 and NEIL2 [1, 28–30].

#### 2.1.2. APE1 Interaction with Other Proteins Associating BER

APE1 is also involved in DNA repair processes by interacting with other components of DNA repair, such as DNA polymerase *β*, proliferating cell nuclear antigen (PCNA), X-ray cross-species complementing 1 (XRCC1), and others, in addition to its own independent biochemical activity. Various proteins interacting with APE1 are involved in the BER process by stimulating the protein's activity, recruiting other factors, or enhancing the AP endonuclease activity. Interactions between APE1 and DNA polymerase *β* or flap endonuclease 1 (FEN1) induce the removal of the remaining abasic site at the 5′-end of the cleavage site after excision of the AP site by APE1 [31, 32]. DNA polymerase *β* synthesizes new matching nucleotides and then removes 5′-end of APE1 products to proceed to the last step of BER, whereas FEN1 merely cleaves downstream of the DNA nick generating multiple gaps for DNA polymerases [28, 33, 34]. Additionally, interaction between FEN1 and PCNA enhances the excision reaction as well as coordinating long-patch BER [32]. Previous research in our group demonstrated that the interaction of growth arrest and DNA damage inducible, alpha (Gadd45*α*) with PCNA plays a critical role in modulating BER activity by affecting the involvement of APE1 [35, 36]. In addition, our group showed that the interaction between Gadd45*α* and both APE1 and PCNA is induced by p53-mediated BER activity with organic selenium compounds [37]. Physical association of APE1 with XRCC1 is strongly implicated in the processing and repair of ssDNA breaks in mammalian cells [38, 39], leading to reinforcing of AP endonuclease and 3′-phosphodiesterase activity [40]. Indeed, heat-shock protein 70 (HSP70) is one of the heat shock protein members involved in general stress response and can be induced by heat shock, oxidation, and other stresses [41]. Additionally, several reports demonstrated that a direct interaction between APE1 and HSP70 strongly stimulated the endonuclease activity of APE1 at AP site, as evident by APE assay with varying amounts of HSP70 [42–44]. This suggested that HSP70 might be involved in the protective mechanism (so called BER) against oxidative stress. Altogether, the multiple contributions of APE1 to DNA repair activity in processing BER are evidence that this protein is crucial for maintaining genetic integrity and cellular existence.

### 2.2. Redox Activity of APE1

#### 2.2.1. Principal of Redox Activity in APE1

Along with the repair activity of APE1, this protein is a redox factor that regulates various transcription factors [17, 45]. The modulation of transcription factors activity is controlled by the redox state of reactive Cys residues both in the DNA-binding domain of some transcription factors and in the N-terminus domain of APE1. APE1 is a unique redox factor because of the lack of C-X-X-C motif which exists in most redox regulatory factors, such as thioredoxin, and because of adequate position of Cys residues allowing the formation of disulfide bonds in redox process [46, 47]. It has been reported that the Cys 65 residue in APE1 is crucial for redox-activation [6, 48], in spite of the buried location of Cys 65 making it poorly accessible by other proteins [49]. In thiol-mediated redox reactions, a Cys 65 residue of APE1 serves as the nucleophilic residue to form disulfide bonds between APE1 and the target transcription factor causing a conformational change of APE1 [46, 50]. After the mixed disulfide bond formation, another Cys residue in APE1, Cys 93, acts as a resolving residue for reduction of the target protein resulting in the oxidized redox factor [46].

#### 2.2.2. Regulation of Transcription Factors by Redox Regulation

APE1 has been identified as a modulator of DNA-binding activity of various redox-sensitive transcription factors including both ubiquitous (AP-1, Egr-1, NF-*κ*B, p53, CREB, and HIF-1*α*) and tissue-specific (PEBP-2, Pax-5 and Pax-8, and TTF-1) by regulating the redox state [46, 51–60]. Among these transcription factors affected by the redox activity of APE1, AP-1 and p53 have been reported to be involved in carcinogenesis [46]. Activator protein-1 (AP-1), which is involved in cellular proliferation, differentiation, and apoptosis, was first shown to have regulated DNA binding activity caused by APE1 because of a reducing Cys residue in the AP-1's DNA binding domain [51, 61]. APE1 enhances the DNA-binding activity of AP-1 by promoting its dimerization by regulating redox of the basic DNA binding domain of c-Jun and C-Fos [61]. APE1 also controls DNA binding activity of p53 by reducing it which is a transcriptional regulatory protein and a critically crucial tumor suppressor [55, 62]. Although p53 appears to have a redox-independent mechanism, its redox activation can be induced by a general redox factor because of low binding affinity to DNA in oxidized p53 [62, 63]. p53 affects many DNA repair pathways which depend on genotoxic stress; obviously APE1 plays a crucial role in the regulation of DNA repair [64, 65].

### 2.3. Other Functions of APE1

#### 2.3.1. RNA Metabolism and APE1

The relation between APE1 and RNA metabolism has been widely investigated recently. APE1 can degrade a basic site in RNA, which suggests that APE1 plays a role in RNA quality control by removing damaged RNA templates [97, 98]. Barnes and colleague documented that human APE1 is able to cleave an RNA molecule at the coding region determinant of the c-myc mRNA [99]. This RNA modulation activity of APE1 is controlled by the N-terminal amino acids, which indicate that APE1 is involved in the posttranslational regulation, even though this mechanism is until now not sufficiently clear.

#### 2.3.2. Immunoglobulin Modification by APE1

A newly identified role of APE1 is in the antibody class switch recombination (CSR). Earlier study reported a slight decline of CSR in an APE1-haplodeficient mouse model [100]. More recently, direct evidence shows a critical role of APE1 in CSR in mouse B cell line [101]. However, there are many discordant studies about immunoglobulin CSR, and further investigations are necessary to identify exact mechanisms.

## 3. APE1 in Human Pathologies

Since APE1 is well known to have AP endonuclease and redox activities [102], and more recently discovered functions, such as affecting RNA metabolism [97], the importance of APE1 in human pathologies is not unexpected. Since the role of APE1 in human pathologies including cancer has been recently reviewed in detail [1, 13, 103], we will only briefly discuss the issue.

### 3.1. Cancer

APE1 has received significant attention as an attractive target for pharmacological treatment in some cancer types. Alteration of APE1 expression and localization is, in particular, a well-established common feature in different neoplastic diseases [31], suggesting that APE1 may have prognostic and/or predictive significance in cancer. Fishel et al. [104] demonstrated that using siRNA technology that reduced levels of APE1 dramatically slows the growth of ovarian cancer cells, both* in vitro* and* in vivo*. In addition, a decrease in APE1 protein levels resulted in pancreatic cancer cell growth inhibition, increased apoptosis, and altered cell cycle progression [105].

APE1 localization is regulated, though the mechanism by APE1 compartmentalization is not clearly understood. In general, APE1 is preferentially expressed in nuclear, but the nuclear, cytoplasmic, and nuclear/cytoplasmic expressions were found in several types of cancer including epithelial ovarian cancer [106], thyroid carcinomas [107], and non-small-cell lung cancer [108].

The dysregulation of APE1 expression is considered as a potential marker to predict the sensitivity of the tumor against radio- or chemotherapy. An inverse relationship between radiosensitivity and the levels of APE1 was reported in cervical carcinoma [109], colorectal cancer [110], and pancreatic cancer [111]. Moreover, Robertson et al. [112] have suggested that the overexpressing APE1 observed in human testicular cancer might lead to cellular protection from bleomycin treatment.

APE1 polymorphism is also important in cancer susceptibility and development. Eighteen polymorphisms in APE1 have been reported [113], but the most extensively studied polymorphism is D148E (rs1130409) [114, 115]. Although the D148E polymorphism of APE1 is frequent and does not impart a reduction in AP endonuclease efficiency [114], numerous reports have suggested that D148E polymorphism is associated with increased susceptibility to colorectal, gastric, and prostate cancer, as well as cutaneous melanoma [116–120]. The APE1 −656T > G in the promoter region is another widely studied polymorphism which is suggested to influence the gene expression at the transcriptional level. The functionally significant −656T > G polymorphism of APE1 contributes to the susceptibility to breast and cervical cancers [121, 122]. In contrast, the −656T > G polymorphism shows a decreased risk of bladder cancer [123]. The polymorphism of APE1 D148E and −656T > G has also been extensively studied in lung cancer [80, 124–127], but confusing results demand further elucidation.

### 3.2. Neuropathology

APE1 is highly expressed in the central nervous system (CNS), albeit it varies in different cell types and regions of the human brain [128]. Vasko et al. [129] demonstrated that decreased APE1 level in primary rat hippocampal or sensory neuronal cell caused the inhibition of cell viability and enhanced apoptosis and DNA damage under hydrogen peroxide treatment. In particular, the inherited defect in DNA repair pathways was suggested as one of the main causes for diverse neurological disorders in human.

Numerous studies have suggested chronic oxidative stress as a cause of neurodegenerative diseases, such as Alzheimer's disease (AD), Parkinson's disease (PD), and amyotrophic lateral sclerosis (ALS), implying the potential importance of DNA repair genes, including APE1, as risk factors. AD causes functional impairments through neuron losses in the cerebral cortex, whereas PD is characterized by motor deficits and cell death in the substantia nigra. ALS is a debilitating disease that causes muscle weakness and atrophy throughout the body due to the degeneration of the upper and lower motor neurons.

The hippocampus and surrounding temporal cortex of patients with AD showed an increased expression of APE1 levels relative to matched controls in senile plaques and plaque-like structures [130]. Recently, Marcon et al. [131] have reported that an increased nuclear expression of APE1/Ref-1 in neuronal and glial cells of the cerebral cortex in both familial and sporadic AD brains might be associated with the cellular adaptive response to the oxidative stress condition. In contrast, the frontal cortical levels and activity of APE1 were significantly lower in 11 patients with sporadic ALS than in controls [132].

Recently, Gencer et al. [133] have suggested that APE1 genetic variant (D148E) might be a risk factor by causing the loss of dopaminergic cells in the substantia nigra and locus caeruleus, and, ultimately, the development of PD. In addition, APE1 mutations including the missense variants L104R, E126D, D148E, D283G, and G306A were found in eight of 11 patients with ALS and familial ALS [134]. Although the correlations between APE1 malfunction and various neuropathologies have been widely accepted, few studies have focused on the mechanism of the protective effect of APE1 as a target for future therapeutic development.

### 3.3. Age-Associated Disorders

The accumulation of oxidative DNA damage, which leads to cellular malfunctions, has been considered as a main cause of aging [135]. Consequently, it has been suggested that a decrease of the repair capacity to remove oxidatively damaged DNA correlates with age-related disease. Indeed, Intano et al. [136] showed that an 85% decline in BER activity was observed in brain nuclear extracts and a 50% decrease in liver nuclear extracts prepared from old mice compared with 6-day-old mice. Recently, Swain and Rao [137] have demonstrated that the APE1 activity in rat brain decreased significantly with age. Since APE1 decreases intracellular ROS by inhibiting rac1 regulated NAD(P)H oxidase [138], its expression change can also affect the aging. The gene expression of mRNA and APE1 enzyme decreased with age in the lenses of rats, resulting in a decrease in the repair capabilities and an accumulation of damaged DNA [139]. In addition to the alteration of APE1 expression and activity, the age-dependent redistribution of APE1 in the nucleus and mitochondria was found in mouse liver [140]. Moreover, recent study has reported that APE1 was an essential factor stabilizing telomeric DNA and its deficiency was associated with telomere dysfunction and segregation [141], suggesting the deeper molecular mechanism of APE1 in aging because dysregulated or shorter telomeres are thought to be a cause of aging.

### 3.4. Other Diseases

Other than in the disorders mentioned above, APE1 deregulation has also been demonstrated in other pathologies. APE1 polymorphism significantly increased the risk of myocardial infarcts [142] and ulcerative colitis [143]. Moreover, Jiang et al. [144] have demonstrated that the inhibition of APE1 redox activity using a small molecule inhibitor APX3330 blocked retinal angiogenesis* in vitro* and* in vivo*. The inhibited APE1 redox function blocks the ability of HIF-1a to bind to various downstream target promoters including angiogenic molecules [145, 146]. This evidence suggests that APE1 may have potential as a therapeutic target for antiangiogenesis treatment in retinal neovascular disorders.

## 4. APE1 as a Prediction Marker of Environmental Carcinogenesis Risk

Almost all diseases result from complex interactions between an individual's genetic make-up and environmental factors. There is great significance of gene-environment interaction in the development of diseases, especially lethal diseases including cancer. In particular, several reports suggested that genetically based variability (silencing, polymorphism) of the proteins involved in cancer metabolism may influence susceptibility to environmental carcinogens [147]. Here, we summarize the interference of APE1 expression and function with environmental factors including heavy metals, smoking, and radiations, with emphasis on the gene-environment interaction studies focused on APE1 in terms of human pathologies (Table 1).

### 4.1. Heavy Metals

#### 4.1.1. Arsenic

Arsenic (As) is a common environmental contaminant that enters humans through drinking water. In 2002, the International Agency for Research on Cancer (IARC) has classified As in drinking water as a group 1 human carcinogen [148]. Besides the induction of different types of cancer (skin, bladder, liver, kidney, and lung) [149, 150], sublethal exposure to As can cause severe human health problems such as diabetes [151] and neurological diseases (Alzheimer and Parkinson's) [152, 153]. While epidemiological studies have clearly demonstrated the harmful effects of As with respect to the induction of human diseases, the mechanisms of toxicity remain largely unknown except ROS generation [154].

It is known that As can affect both the endonuclease and the redox functions of APE1 to increase oxidative stress and inhibit DNA repair. In particular, APE1 activity is affected indirectly by As through the changes in transcription levels [66, 68]. Sykora and Snow [69] have reported that APE1 mRNA exhibited significant dose-dependent downregulation in response to low, physiologically relevant doses of As. Similarly, through the analysis of gene expression profiling by As exposure in human lung cells, the exposure to sodium arsenite for 4 hours decreased APE1 mRNA level [67]. In contrast, several reports found the stimulated APE1 expression and activity in cultured cells exposed by submicromolar As doses [69, 70, 155].

APE1 is associated with the oxidative biomarker, 8-hydroxy-2′-deoxyguanosine (8-OHdG), which can be induced by As exposure. The repair of 8-OHdG lesions requires both apurinic/apyrimidinic endonuclease (APE1) and human 8-oxoguanine glycosylase (hOGG1) [156]. Indeed, the polymorphisms (D148E) in the APE1 gene led to the decreased repair ability of oxidative DNA damage [157] and affected the urinary 8-OHdG concentrations in Vietnamese exposed to As [71]. Moreover, Caucasians showed higher mutant frequencies in APE1 than those of African and Asian populations in response to As. Among Asian populations, the Bangladeshi population showed relatively higher mutant allele frequencies of the APE1 D148E [71]. Another study on an As-exposed population conducted in Bangladesh explored some polymorphisms in BER genes, providing evidence that APE1 was related to As-induced skin lesions [72]. These accumulated data emphasize the importance of the combination of APE1 polymorphism with heavy metal exposure in terms of human disease development.

#### 4.1.2. Cadmium

Cadmium (Cd) is a hazardous heavy metal that induces cytotoxicity and carcinogenicity upon persistent environmental exposure. In 1993, the International Agency for Research on Cancer (IARC) classified Cd compounds as group 1 carcinogens in humans [158]. Chronic exposure to Cd causes a wide range of toxicity-related diseases, including cancer in the lung, prostate, kidney, liver, and testis in humans and other mammals [159, 160]. Although the molecular mechanisms of toxicity and carcinogenicity of Cd remain poorly understood, two commonly suggested mechanisms for toxicity are the induction of oxidative DNA damage and the inhibition of DNA repair [160–162]. BER triggered by oxidative DNA damage is one of the important target mechanisms against Cd genotoxicity [163]. In particular, Cd inhibits the initial steps of BER, including the removal of AP site by APE1 endonuclease activity [164].

McNeill et al. [73] reported that Cd selectively inhibited APE1 endonuclease activity in whole-cell extracts but had no significant effect on single nucleotide gap filling, 5′-flap endonuclease, and nick ligation activities. In addition, recent evidences have demonstrated that Cd also impaired APE1 mRNA level as well as its activity [74, 75]. With respect to the potential role of tumor suppressor p53 in mechanism study for cadmium-induced inhibition of APE1 activity, the genotoxic stress was induced by Cd activated p53 and led to a significant downregulation of APE1 by p53 in HCT116 p53^+/+^ cells [74]. However, another study has found that* in vivo* treatment of human cells with Cd at sublethal concentrations had no effect on the APE1 activity [76]. Although they suggested that the intracellular concentrations of free Cd do not reach the levels required for the inhibition of APE1 due to the complexity of Cd within the cells [76], further studies are needed to fully demonstrate the effects of Cd with respect to APE1 activity.

#### 4.1.3. Lead

Lead (Pb) exposure is hazardous to human health because it is widely distributed and persists in the environment. International Agency for Research on Cancer (IARC) classified it as possible human carcinogen (group 2B) [165] and its inorganic compounds as probable human carcinogens (group 2A) [166]. A number of human epidemiological studies and animal experiments have reported the harmful effects of Pb when it causes serious damage to bone, kidney, lung, nervous system, and red blood cell function [167, 168]. Although the induction of oxidative stress, the inhibition of DNA repair, and the formation of DNA/protein crosslink have been suggested as main toxic mechanisms of Pb exposure [169], the results are still conflicting in terms of promoting genotoxicity [170].

The alterations of APE1 level and activity may be a potential target against Pb genotoxicity. Pb inhibited the APE1 endonuclease activity of whole-cell extracts in a concentration-dependent manner with selective inactivation [73, 171]. In addition, McNeill et al. [171] have demonstrated a dose-dependent accumulation of AP sites as well as inhibition of APE1 activity in AP site incision under Pb exposure, implying an underlying mechanism by which Pb promotes cocarcinogenesis. On the other hand, Scortegagna and Hanbauer [77] have reported the enhanced APE1 accumulation in the nucleus in response to Pb treatment. In fact, oxidative stress can increase the expression of APE1, which functions against the genotoxic responses in diverse mammalian cells [70, 172, 173]. Interestingly, Pb-exposed workers showed the induced APE1 D148E polymorphism on T-cell receptor mutation frequency, suggesting a role for APE1 polymorphism as a susceptibility biomarker in biomonitoring by occupational Pb exposure [78].

### 4.2. Smoking

Cigarette smoke contains many carcinogens including heavy metals and has been clearly identified as a direct cause of lung cancer [174]. Cigarette smoke can induce various types of DNA damage [175]; consequently, individuals with reduced DNA repair ability would be expected to accumulate more carcinogen-induced DNA adducts in their tissue [176]. Indeed, lung cancer patients, mainly smokers, may have a lower capacity of DNA repair compared with healthy subjects and this can be suggested as a target mechanism for risk prediction of lung cancer associated with smoking [177–179].

Several studies have reported that cigarette smoke condensate (CSC) increased expression of adenomatous polyposis coli (APC) and blocked long-patch base excision repair (LP-BER) in human breast epithelial cells [180, 181]. In addition, benzo[*α*]pyrene, a major constituent of CSC, enhanced APC-mediated accumulation of abasic DNA lesions, leading to increased neoplastic transformation of breast epithelial cells [182]. Smoking also leads to brain disorder because, as la Maestra et al. [79] demonstrated, cigarette smoke altered BER by decreasing APE1 expression in the hippocampus and cerebellum of neonatal mice.

A number of epidemiological studies have evaluated the relationship among polymorphisms of APE1, smoking, and the risk of lung cancer. Several reports have suggested that APE1 genotypes were not major determinants of lung cancer susceptibility among smokers [85, 86], while several authors have asserted that APE1 polymorphisms such as −656T > G located in the promoter region and D148E could modify the risk of developing lung cancer attributable to cigarette smoking exposure [80–83]. With regard to bladder cancer, Huang et al. [84] have found that the gene-environment interaction between smoking and APE1 D148E polymorphism was statistically significant for the risk of the cancer. In contradiction, Terry et al. [87] found no overall association between APE1 genotype and bladder cancer risk. Although the precise biological mechanisms of the interaction of APE1 phenotype and smoking still need to be clarified, these studies provide potential evidence of gene-environment interactions between APE1 polymorphisms and smoking in cancer development.

### 4.3. UV and Other Radiations

Ultraviolet (UV) can induce DNA repair process directly or indirectly through the photoproducts generation or the ROS accumulation. Indeed, Izzotti et al. [88] reported that the expression of genes involved in oxidative stress response and DNA repair mechanism (including APE1) were increased in the skin of SKH-1 hairless mice exposed to UVC. UVA is also a critical component of solar radiation that has been implicated in photocarcinogenesis. UVA irradiation induced APE1 polymorphisms on various chromosome aberration types and relocalization of this protein to nuclear speckles against oxidative stress [89, 90].

Ionizing radiation (IR) is a widely used therapy for treating various types of cancers. However, it causes damage to normal cells of living tissue vicinity to tumors, resulting in mutation, radiation sickness, cancer, and ultimately death [183, 184]. Several reports demonstrated that IR induced APE1 polymorphism through the analysis of blood in breast cancer patients or occupationally exposed workers [91–93]. In particular, diagnostic X-rays increase the risk of developmental abnormalities and cancer in the exposed individuals [185–187].* In vitro* findings revealed that the mammalian cells exposed to X-rays caused change in the expression and activity as well as polymorphism of APE1 [89, 94–96]. Taken together, these studies suggest that APE1 can be a vital marker for risk prediction upon exposure of physical carcinogens in addition to chemical carcinogens.

## 5. Concluding Remarks

**Figure 2:**
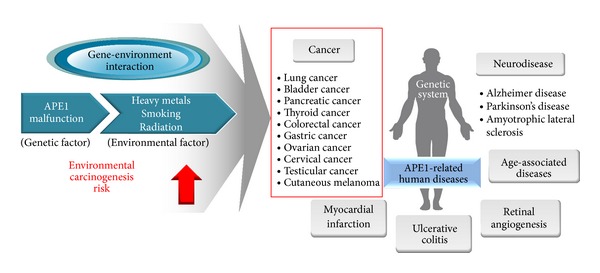
Scheme showing the potential of APE1 as an important modulator in human diseases. APE1 abnormality is known to be associated with the induction of various lethal diseases including cancer. In particular, gene-environment interaction between APE1 malfunction and several environmental factors increases the carcinogenic risk, leading to cancer development. Thus, APE1 can be suggested as one of the potential markers for risk prediction of environmental carcinogenesis.

There is no doubt of critical roles of APE1 in maintenance of genomic stability against endogenous and exogenous stresses. APE1 possesses powerful biological capacities in various cellular processes such as DNA repair, redox regulation, cell cycle, and RNA modulation. Because of the widely known APE1 multifunction, there are many reports that deal with the importance of APE1 in human disease developments. In particular, previous cell-based, animal model, and epidemiological studies suggested that the genetic variation (e.g., polymorphism) of APE1 as well as the alteration of APE1 localization, expression, and activity can be possible causes of lethal disease, including cancer (Figure 2). Although APE1 is overexpressed in many tumors and its enhanced nuclear levels correlate with reduced sensitivity to anticancer drugs, our review emphasizes the possibility of APE1 of relatively low level as a protector in human diseases induced by environmental factors under normal conditions, not under the abnormal conditions of the APE1 level in the cancerous predisposition.

Since several studies showed that APE1 malfunction enhances the risk of disease development when a person is exposed to harmful substances, we attempted to suggest APE1 as an important modulator in human disease caused by various environmental factors considering gene-environment interactions. Heavy metals such as arsenic, cadmium, and lead are widespread environmental pollutants and cause detrimental health effects including cancer, even at low doses. Although the molecular mechanisms of toxicity and carcinogenicity of these heavy metals remain poorly understood, commonly suggested mechanisms involve induction of oxidative DNA damage and the inhibition of DNA repair. It is well known that these heavy metals can change the expression and activity of APE1 and such APE1 malfunction is a vital factor related to severe human diseases. Nevertheless, as shown in Table 1, since the study on gene-environment interaction between APE1 and heavy metal in human pathologies is almost nonexistent, further studies are positively necessary. With regard to smoking of cigarette and radiation exposure, though the clear evidences of correlation among APE1, environmental factor, and related diseases are still required, the accumulated evidences suggest that APE1 may be a promising marker for risk prediction of environmental diseases including cancer.

## Figures and Tables

**Table 1:** Interference of APE1 upon environmental carcinogen exposure using *in vitro *and* in vivo* mammalian models and human population samples.

Environmental factor	Subject	Dose	Effect on APE1	Reference
**Heavy metal**				
Arsenic	Human skin cell line	0.005–5 *μ*M	Decrease of APE1 mRNA expression	[66]
	Human lung cell line	5 *μ*M	Decrease of APE1 mRNA expression	[67]
	Human skin cell line	0.1–5 *μ*M	Increase of APE1 protein expression (short-term)	[68]
		0.1 or 0.5 *μ*M	Decrease of APE1 protein expression (long-term)	
		>1 *μ*M	Dose-dependent decrease of APE1 mRNA expression	
	Human lung and skin cell lines	<1 *μ*M	Increase of APE1 protein expression	[69]
		5–100 *μ*M	Dose-dependent increase of APE1 mRNA expression	
	Mouse embryo cell line	10–75 *μ*M	Dose-dependent increase of APE1 protein expression	[70]
		10–100 *μ*M	Dose-dependent increase of APE1 activity	
	Human population	—	Induction of APE1 polymorphism (D148E)	[71]
	Human population	—	Induction of skin lesions with APE1 polymorphism (D148E)	[72]∗
	Human kidney cell line	100 *μ*M	No effect on APE1 activity	[73]

Cadmium	Human colon cell line	>10 *μ*M	Decrease of APE1 mRNA expression	[74]
		>25 *μ*M	Decrease of APE1 activity	
	Human kidney cell line	100 *μ*M	Decrease of APE1 activity	[73]
	Human population	—	Decrease of APE1 mRNA expression	[75]
	Human breast and cervix cell lines	20–80 *μ*M	No effect on APE1 protein expression and activity	[76]

Lead	Mouse brain cell	10 *μ*M	APE1 accumulation in nucleus	[77]
	Hamster ovary cell line	0.5–500 *μ*M	Dose-dependent accumulation of AP sites and decrease of APE1 activity	[4]
	Human kidney cell line	100 *μ*M	Decrease of APE1 activity	[73]
	Human population	—	Induction of APE1 polymorphism (D148E)	[78]
	Human population	—	No effect on APE1 mRNA expression	[75]

**Smoking**				
	Swiss ICR albino mice	119, 292, 438, 631 mg/m^3^ (TSM^§^)	Decrease of APE1 protein expression in brain tissue	[79]
	Human population	—	Induction of lung cancer with APE1 polymorphism (D148E and −656T > G)	[80–83]∗
	Human population	—	Induction of bladder cancer with APE1 polymorphism (D148E)	[84]∗
	Human population	—	No effect on the induction of lung cancer with APE1 polymorphism (D148E)	[85, 86]
	Human population	—	No effect on the induction of bladder cancer with APE1 polymorphism (D148E)	[87]

**Radiation**				
Ultraviolet	SKH-1 hairless mice	5 days a week, 9 h a day at 10,000 lx (UVC)	Increase of APE1 mRNA expression	[88]
	Human lymphocyte	4 J/m^2^ (UVA)	Induction of APE1 polymorphism (D148E)	[89]
	Human cervix cell line	0.2 J/cm^2^ (UVA)	Induction of APE1 relocalization to nuclear speckles	[90]

Ionizing radiation	Human population	—	Induction of APE1 polymorphism (D148E)	[91]
	Human population	—	Induction of breast cancer with APE1 polymorphism (D148E)	[92]
	Human population	—	Induction of APE1 polymorphism (D148E)	[93]
	Human lung cell line	200 cGy/min (X-ray)	Increase of APE1 protein expression	[94]
	Human lung cell line	4, 16 Gy (X-ray)	Increase of APE1 protein expression	[95]
	Human blood culture	80 cGy/min (X-ray)	Induction of APE1 polymorphism (D148E)	[89]
	Human lymphocyte	1 Gy/min (X-ray)	Induction of APE1 polymorphism (D148E)	[96]

∗It represents study of gene-environment interaction; ^§^TSM is abbreviation of total suspended matter in average after burning cigarette.
